# Myasthenia

**Published:** 1900-03-10

**Authors:** 


### MYASTHENIA.

In a clinical lecture delivered at the Queen's Square Hospital, Dr. Buzzard described the condition known as Myasthenia Gravis Pseudo-paralytica, a condition of considerable interest, of which only about 70 cases in all have as yet been published by observers in different parts of the world. So far as symptoms go this curious affection is characterised by a progressive muscular weakness, a paresis, affecting different muscles in different cases, sometimes tending to recovery, at others ending in death from respiratory disturbances such as are met with in bulbar paralysis, but without any apparent pathological changes in the medulla. In most cases there is considerable variation in the extent and degree of the muscular debility from day to day, and what is especially peculiar is the electrical reaction, the muscles quickly becoming fatigued, so that they cease to respond to faradism. As was shown in the course of the lecture on stimulating the biceps by the faradic current, strong and rapid supination of the forearm was produced with some flexion of the elbow, but gradually, as the applications were rapidly repeated, the movements diminished in extent, until after about a minute none were visible. After about half a minute's rest, however, vigorous contraction could be produced again. This reaction, the rapid production of fatigue paralysis, might be called the myasthenic reaction. The rapid production of exhaustion of certain muscles by use is then the most marked symptom. Thus, in one case, after walking for half an hour or so, the legs began to feel weak, so that the patient had to stop and rest, but after resting a few minutes she could go on again, but not so far as before. When her arms became affected she became unable to write; after writing a few lines the fatigue in her hand obliged her to stop. Then she began to speak through her nose, particularly when tired, and especially towards evening her voice would gradually become weaker until she became quite unable to speak for a minute or two. As to what is the nature of this disease it seems impossible at present to say anything definite. By some it is supposed to be due to the presence of a toxin in connection, perhaps, with disordered metabolism, and the strange remissions and exacerbations which the disease exhibits certainly point in that direction. As to locality, the balance of probability points to the cells of the motor cortex as being the seat of the morbid process. There is, no doubt, a danger of these being mistaken for examples of hysteria, but the presence of the "myasthenic reaction" ought to prevent such an error, one which would be very serious, for the prognosis is very grave, and death often occurs rapidly. In regard to treatment there is not much to be said, except to give warning against the production of fatigue of the muscles of respiration and deglutition, a fatigue which may readily induce death. Thus, the application of the faradic current to these muscles is to be avoided, and it is important not to attempt to feed by the tube. Ordinary swallowing may be exhausting, but the fatigue of the respiratory muscles caused by the choking set up by passing the tube may be still more serious—may, indeed, cause death.

